# Antiperovskite Li_3_OCl Superionic Conductor Films for Solid‐State Li‐Ion Batteries

**DOI:** 10.1002/advs.201500359

**Published:** 2016-02-02

**Authors:** Xujie Lü, John W. Howard, Aiping Chen, Jinlong Zhu, Shuai Li, Gang Wu, Paul Dowden, Hongwu Xu, Yusheng Zhao, Quanxi Jia

**Affiliations:** ^1^ Center for Integrated NanotechnologiesLos Alamos National LaboratoryLos AlamosNM87545USA; ^2^ High Pressure Science and Engineering CenterUniversity of NevadaLas VegasNV89154USA; ^3^ Department of Chemical and Biological EngineeringUniversity at BuffaloThe State University of New YorkBuffaloNY14260USA; ^4^ Earth and Environmental Sciences DivisionLos Alamos National LaboratoryLos AlamosNM87545USA

**Keywords:** antiperovskite phase, solid‐state batteries, superionic conductor, sustainable chemistry, thin films

## Abstract

**Antiperovskite Li_3_OCl superionic conductor films** are prepared via pulsed laser deposition using a composite target. A significantly enhanced ionic conductivity of 2.0 × 10^−4^ S cm^−1^ at room temperature is achieved, and this value is more than two orders of magnitude higher than that of its bulk counterpart. The applicability of Li_3_OCl as a solid electrolyte for Li‐ion batteries is demonstrated.

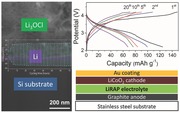

Electrical energy storage becomes increasingly more important. It was reported by McKinsey that the potential economic impact of improved energy storage could be $90 to $635 billion per year by 2025.^1^ Although Li‐ion batteries (LIBs) are powering billions of portable electronic products as well as hybrid and electric vehicles, the safety issues related to the corrosive and flammable liquid electrolytes in current commercialized LIBs must be addressed.^2^, ^3^, ^4^ Replacing liquid electrolytes with solid‐state inorganic electrolytes has been considered as the ultimate solution to address the safety issues. In addition, the solid‐state electrolytes can be readily integrated with a metallic lithium anode, allowing solid‐state LIBs with potentially high energy densities. Thus far, solid‐state electrolytes have not been widely used in the commercial products because their ionic conductivities are not high enough to meet the requirements for high‐performance Li batteries.^5^, ^6^, ^7^ Hence, it is extremely important to develop advanced solid‐state electrolytes with high ionic conductivity and good compatibility with cathode and anode materials for all‐solid‐state LIBs.

Many inorganic Li‐ion‐conducting materials such as lithium nitrides (e.g., Li_3_N), lithium phosphorous oxynitrides (e.g., Li_2_PO_2_N), lithium sulfides (e.g., Li_3_PS_4_, Li_3_SnS_4_), and garnets (e.g., Li_7_La_3_Zr_2_O_12_) have been explored as solid electrolytes and have shown potential applications in prototype solid‐state Li batteries.^8^, ^9^, ^10^, ^11^, ^12^, ^13^, ^14^ However, the relatively low ionic conductivities of solid electrolytes in comparison with liquid electrolytes, as well as the interface‐related issues, have hindered the progress in the development of all‐solid‐state LIBs. Recently, lithium‐rich antiperovskite (LiRAP), Li_3_OX (X = Cl, Br, etc.), has been developed as a promising solid electrolyte.^15^, ^16^, ^17^, ^18^, ^19^, ^20^ This class of electrolytes accommodates a large number of Li ions in the crystal lattice and the Li ions can move easily by introducing certain amounts of crystal defects, such as Li^+^ and Cl^–^ vacancies. The most important advantages of the LiRAP electrolyte include (1) high ionic conductivity and low energy barrier for Li transport, (2) low electronic conductivity (Li_3_OCl with a band gap exceeding 5 eV^16^, ^17^) with minimum self‐discharge for long shelf life, (3) wide electrochemical working windows beyond 5 V compatible with high‐potential cathodes, (4) stable operation at high temperatures up to 275 °C, and (5) environmental friendliness.

The solid‐state electrolytes also enable the fabrication of thin and flexible batteries, which are urgently needed in burgeoning applications such as wearable electronics and implantable medical devices.^21^, ^22^, ^23^ Research into thin film batteries has significantly grown in recent years to accommodate this increasing demand. To construct a thin‐film LIB, it is necessary to fabricate all components such as anode, electrolyte, and cathode materials into multilayered films with good structural integrity using appropriate technologies. The growth of solid electrolyte films with desired structure is one of the key steps to ensure high ionic conductivity and controllable interfaces. We have recently reported the preparation of Li_3_OCl film by pulsed laser deposition (PLD), and the ionic conductivity was notably enhanced in comparison with that of its bulk counterpart.^19^ In that work, we used the stoichiometric Li_3_OCl compound as the target, where the conditions to prepare target are critical for obtaining high performance Li_3_OCl films. Furthermore, the process to prepare the Li_3_OCl target is energy/time consuming (>300 °C for at least 48 h). Even more challenging, the Li_3_OCl compound is very sensitive to atmospheric moisture, which makes it extremely difficult to handle. In this work, we report the use of a new composite target to deposit Li_3_OCl films by PLD. Specifically, we have simply mixed the raw materials of Li_2_O and LiCl and compressed them into a pellet as the target without preformation of Li_3_OCl compound. Such a scheme results in a much more stable target which makes it much easier to handle before, during, and after film deposition. Intriguingly, high crystalline Li_3_OCl films can be readily formed during the PLD process and the ionic conductivity of the films increases substantially to ≈10^−4^ S cm^−1^ at room temperature. Using a LiCoO_2_ cathode and a graphite anode, we for the first time have successfully demonstrated all‐solid‐state thin‐film LIBs with the newly prepared Li_3_OCl films.

For the film growth by PLD, the characteristics of targets are critical for achieving high quality and desired properties of as‐deposited films. The Li_3_OCl compound target is very sensitive to moisture, which is one of the main issues for processing and practical applications of this new material. As a nonequilibrium process, PLD permits the deposition of high‐quality films at relatively low temperatures. Furthermore, the laser‐induced plasma is highly energetic and would be an energy source to facilitate the chemical reactions between the raw materials during film deposition. Taking the advantages of this process, we used a composite target consisting of two phases of Li_2_O and LiCl (1:1 molar ratio) without preformation of Li_3_OCl compound to grow the antiperovskite Li_3_OCl films. This approach is more energy/time‐efficient, and the prepared target is much more stable.

**Figure** **1**a shows the X‐ray diffraction (XRD) pattern of the composite target. Strong diffraction peaks from Li_2_O and LiCl with only a very weak peak from Li_3_OCl (marked as a star) can be observed. This indicates that the as‐prepared composite target is mainly comprised of the raw materials Li_2_O and LiCl. The formation of Li_3_OCl compound from the reaction between Li_2_O and LiCl should be minimal during target preparation. The comparison between the XRD patterns of the composite and fully reacted compound targets is shown in Figure S1 in the Supporting Information. The Li_3_OCl films were deposited on different substrates (stainless steel and Si) by PLD (KrF laser, 248 nm, 30 Hz) under vacuum (1 × 10^−5^ Torr) using the composite target (see experimental details in the Supporting Information). As shown in Figure 1b, the XRD pattern of the PLD film clearly shows the antiperovskite Li_3_OCl phase, where the strongest peak at 32.7° can be indexed as (011) of the cubic perovskite with the space group of *Pm3m*. It should be noted that no peaks belonging to the raw materials of Li_2_O and LiCl can be discerned in the XRD pattern of the film, which implies that LiCl and Li_2_O have fully reacted during the deposition process. This finding confirms that the laser‐induced energetic plasma is highly effective to enhance the chemical reaction between LiCl and Li_2_O to form crystalline Li_3_OCl, which is very hard to achieve using traditional synthetic methods such as solid‐state reaction.

**Figure 1:**
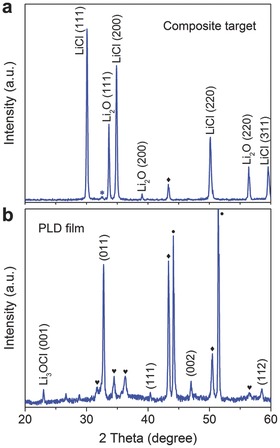
XRD patterns of a) the composite target and b) the Li_3_OCl film deposited using the composite target. Strong peaks of Li_2_O and LiCl with only a very weak peak of Li_3_OCl (110) (star mark) indicate that the composite target is mainly a mixture of raw materials of Li_2_O and LiCl. In the as‐deposited film, sharp peaks of the cubic Li_3_OCl perovskite can be observed without any peak from Li_2_O or LiCl. The symbols of ♥, ♦, and ● indicate the peaks of the ZnO protective layer, Cu sample holder, and stainless steel substrate, respectively.

Electrochemical impedance spectroscopy measurements were conducted in the frequency range from 100 Hz to 4 MHz with a voltage amplitude of 10 mV. The impedance spectrum of the Li_3_OCl film measured at room temperature is presented in **Figure** **2**a. The ionic conductivity was calculated to be 2.0 × 10^−4^ S cm^−1^ at room temperature, which is much higher than those of the ceramic bulk Li_3_OCl (5.8 × 10^−7^ S cm^−1^) and the Li_3_OCl film (0.9 × 10^−5^ S cm^−1^) deposited using a Li_3_OCl compound target.^19^ It is not surprising that the Li_3_OCl film possesses higher conductivity than its bulk counterpart. However, it is intriguing that the films deposited using the composite target show better performance than those deposited using a compound target, which will be further elaborated on later. As the temperature increased to 140 °C, the conductivity value reached 5.9 × 10^−3^ S cm^−1^. The temperature‐dependent ionic conductivities are shown in Figure 2b as a typical Arrhenius plot, which reflects an increase of the conductivity with temperature in an exponential manner described by σ*T = A* × exp*(−E*_a_ */kT)*, where *σ* is the ionic conductivity, *E*_a_ is the activation energy for Li ion transport, *k* is the Boltzmann constant (*k* = 8.617 × 10^−5^ eV K^−1^), and *A* is the pre‐exponential factor. The *E*_a_ derived from the slope of the fitted curve is 0.35 eV, lower than the value for the bulk material of 0.59 eV and similar to the reported value of 0.36 eV for the film deposited using a compound target.^19^

**Figure 2:**
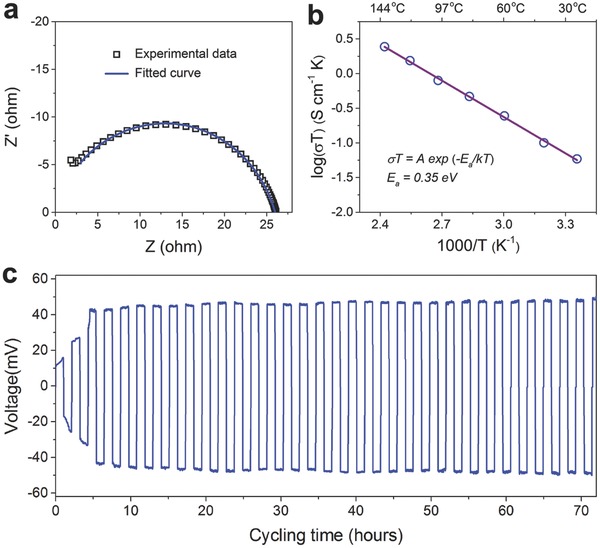
Electrochemical properties of the Li_3_OCl films deposited from the composite target. a) EIS Nyquist plot measured at room temperature (RT) and the corresponding fitted curves. b) Arrhenius plot for Li_3_OCl film measured up to 140 °C. c) Cyclability of the Li/Li_3_OCl/Li symmetric cell. The RT ionic conductivity of the Li_3_OCl films was determined to be 2.0 × 10^−4^ S cm^−1^ and the activation energy *E*_a_ is derived to be 0.35 eV from the slope of the fitted line of log(*σT*) versus 1000/*T*.

As demonstrated by both theoretical arguments and experimental evidences,^17^, ^19^ the depletion of Li^+^ and Cl^−^ in Li_3_OCl crystals is a major mechanism responsible for the higher conductivity of the film compared with that of the bulk material. While the reasons for the different conductivity values of the films deposited using composite or compound targets are unknown, yet are interesting to infer. It is obvious that the Li_3_OCl (011) peak presented in Figure 1b is much sharper than that of the film deposited using a compound target, with their full widths at half maximum being 0.2° and 0.4°, respectively. This indicates larger grain size and higher crystallinity of the Li_3_OCl films deposited using a composite target. It is well known that the resistance of grain boundaries can contribute greatly to the total value. Thus, the larger grain size would reduce the overall resistance, resulting in higher ionic conductivity. Furthermore, theoretical analysis has suggested that the nearest‐neighbor Li‐ion hopping favors along the edge of the Li_6_O octahedron via Li vacancies,^17^ i.e., the (011) planes in the cubic antiperovskite structure. This provides further support for the higher conductivity of the film with larger grain size and a preferentially orientated growth along (011). The decrease of activation energy (0.35 eV for the Li_3_OCl film and 0.59 eV for bulk material) is most likely an indication of structural change (octahedral tilting caused by Li^+^ and Cl^−^ depleting, preferential orientation of the film, etc.) and increase of migration vacancies in the films. The properties of as‐deposited Li_3_OCl films are summarized in Table S2 in the Supporting Information, in comparison with some other reported solid‐state electrolytes.

One of the most prominent advantages of solid‐electrolyte LIBs is their good compatibility with Li metal without the Li dendrite problem commonly encountered in liquid‐electrolyte batteries when Li is used as the anode. The cyclability and compatibility of Li_3_OCl film in contact with metallic Li were evaluated using a symmetric cell of Li/Li_3_OCl/Li by applying a constant direct‐current of 1.0 mA with a periodically changing polarity. Figure 2c shows the voltage profile of the cell cycled continuously for 72 h (1 h per half a cycle). The voltage increases gradually during the first few cycles and then remains approximately unchanged. It has been demonstrated in our previous study that the Li_3_OCl film itself is stable during the cycling process.^19^ While self‐stabilization may happen at the Li/Li_3_OCl interface during the initial cycling. The interface reaches a stable state after certain cycles, as evidenced by the stabilized voltage. In addition, benefiting from the solid‐state feature, the surface of solid electrolytes can be further modified to achieve better compatibility with various electrodes.^12^

The interfacial characteristic between the Li_3_OCl electrolyte film and the Li electrode was further investigated by a scanning electron microscopy (SEM) with an in situ focused ion beam system. The left panel in **Figure** **3** shows a schematic diagram of the multilayer film and the middle and right panels are the cross‐sectional SEM images with different magnifications. From these images, one can see the interfacial contact between Li and Li_3_OCl is relatively smooth.

**Figure 3:**
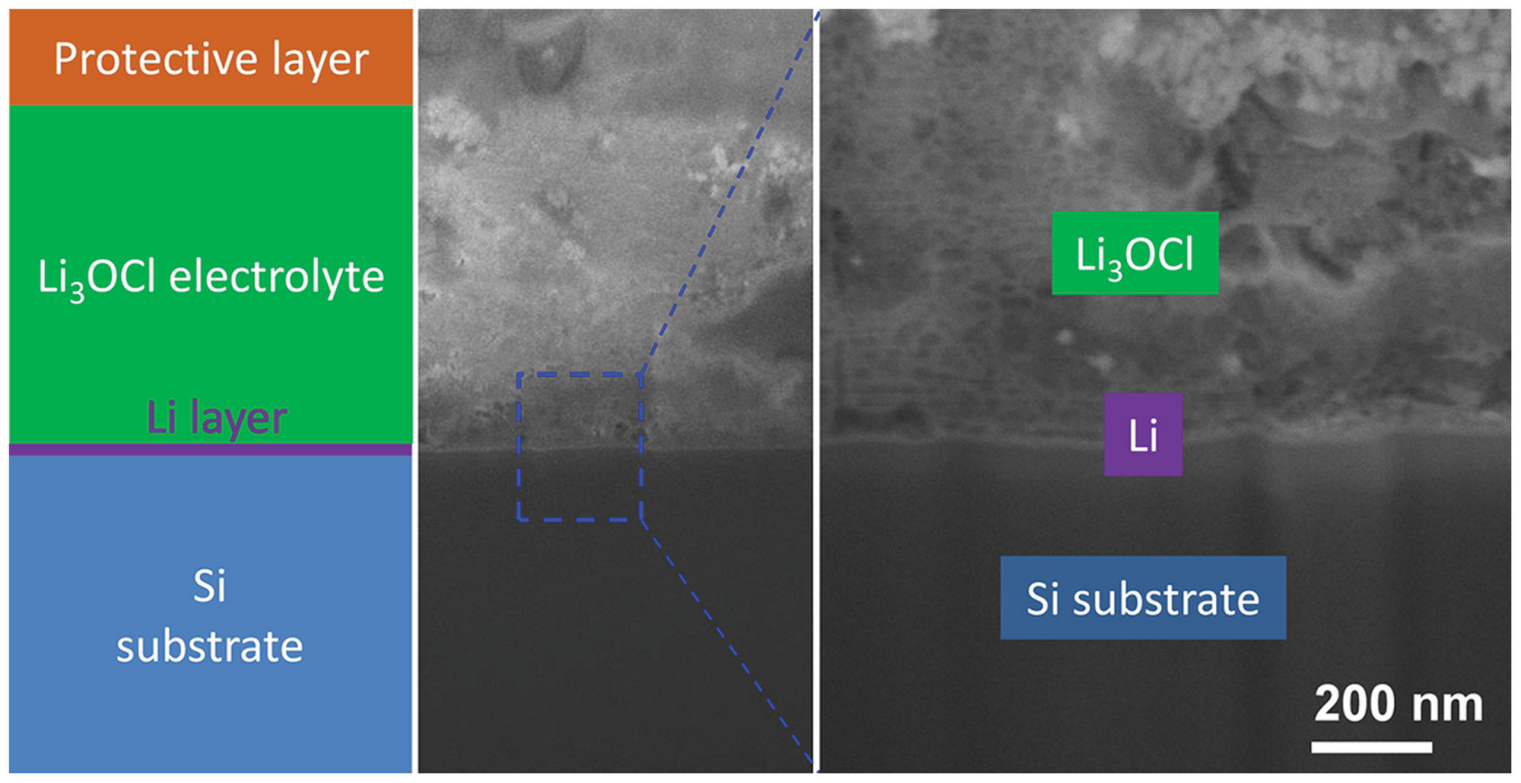
Interfacial characteristic between Li and Li_3_OCl film. The schematic illustration of the multilayer structure (left), the overall cross‐sectional SEM image (middle), and the magnified SEM image of local interfacial structure (right).

To examine the feasibility of Li_3_OCl films as electrolytes in real solid‐state LIBs, we constructed full thin‐film batteries via a layer‐by‐layer deposition without breaking the vacuum. **Figure** **4**a shows the charging–discharging curves of a solid‐state LIB with graphite, Li_3_OCl, and LiCoO_2_ films as the anode, electrolyte, and cathode, respectively. The inset in Figure 4a is a schematic diagram of the multilayer film battery structure. The capacities were calculated from the weight of LiCoO_2_ cathode. The battery has an initial discharge capacity of about 120 mA h g^−1^. The cycling performance of the solid‐state battery is shown in Figure 4b, where the battery exhibits a discharge retention of 55% of the initial capacity after 20 cycles. We noticed that the capacity fading is pretty fast in the initial cycles, which is mainly attributed to the irreversible side reactions in the electrodes and at the interfaces.^24^, ^25^, ^26^ While the continuous capacity loss should be from the moderate sealing condition for our film batteries, where a membrane‐type configuration was used to assemble the film batteries, as illustrated in Figure S2 (Supporting Information). Hence, the battery properties such as stability can be further improved through device fabrication and process optimization such as better sealing and lower moisture environment. The first Coulombic efficiency of the battery is 83%. After the first cycle, Coulombic efficiency is stable at around 95%, slightly lower than that of a typical liquid‐electrolyte LIB. This may result from the lower ionic conductivity of the solid electrolyte than that of the organic liquid electrolytes. However, our results do show that the Li_3_OCl film is a strong candidate as the solid electrolyte for solid‐state film batteries. Moreover, the performance of the antiperovskite electrolyte films can be further improved by optimizing the composition such as cation doping and/or halide mixing.

**Figure 4:**
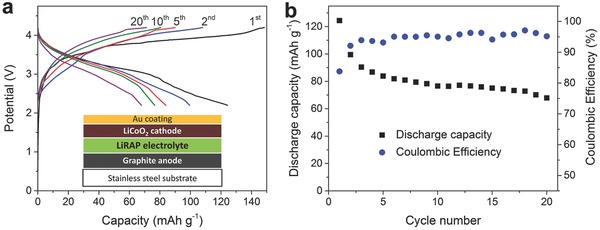
a) Galvanostatic charging–discharging curves of the solid‐state LIB using the Li_3_OCl film as an electrolyte, graphite as an anode, and LiCoO_2_ as a cathode. The inset shows the schematic diagram of the multilayer film battery structure. b) Cycling performance of the solid‐state thin‐film battery. The battery was charged and discharged at 10 mA g^−1^ between 2.2 and 4.2 V. The current density and capacities were calculated from the weight of the LiCoO_2_ film.

In summary, antiperovskite Li_3_OCl superionic conductor films were deposited using a composite target via PLD. The ionic conductivity was determined to be 2.0 × 10^−4^ S cm^−1^ at room temperature, which is improved by more than one order of magnitude in comparison with the film deposited using the compound target (0.9 × 10^−5^ S cm^−1^) and more than two orders of magnitude higher than its bulk counterpart (5.8 × 10^−7^ S cm^−1^). The compatibility of Li_3_OCl film with lithium metal was evaluated by cycling a Li/Li_3_OCl/Li symmetric cell, revealing self‐stabilization during the cycling process. For the first time, the feasibility of Li_3_OCl film as a solid electrolyte in a real battery was demonstrated by examining the full thin‐film LIBs. The all‐solid‐state thin‐film battery showed an initial discharge capacity of 120 mA h g^−1^ and a discharge efficiency of 95% after the second cycle. Our findings provide a kind of promising solid electrolyte film and open a new avenue for the development of next‐generation thin‐film batteries with enhanced safety, reliability, and cyclability.

## Supporting information

As a service to our authors and readers, this journal provides supporting information supplied by the authors. Such materials are peer reviewed and may be re‐organized for online delivery, but are not copy‐edited or typeset. Technical support issues arising from supporting information (other than missing files) should be addressed to the authors.

Supplementary
