# Supplementary material for: Antiperovskite Li3OCl Superionic Conductor Films for Solid‐State Li‐Ion Batteries
Source: Adv Sci (Weinh). 2016 Feb 2;3(3):1500359. doi: 10.1002/advs.201500359 (PMC5067573; doi:10.1002/advs.201500359)
Supplement: Supplementary file 1 — Supplementary [file ADVS-3-1500359-s001.pdf]

Copyright WILEY-VCH Verlag GmbH & Co. KGaA, 69469 Weinheim, Germany, 2016.

ADVANCED SCIENCE
Open Access

Supporting Information

for *Adv. Sci.*, DOI: 10.1002/advs.201500359

Antiperovskite Li_3OCl Superionic Conductor Films for Solid-State Li-Ion Batteries

Xujie Lü, John W. Howard, Aiping Chen, Jinlong Zhu, Shuai Li, Gang Wu, Paul Dowden, Hongwu Xu, Yusheng Zhao,* and Quanxi Jia**

Supporting Information

Anti-Perovskite Li_3OCl Superionic Conductor Films for Solid-State Li-Ion Batteries

Xujie Lü, John W. Howard, Aiping Chen, Jinlong Zhu, Shuai Li, Gang Wu, Paul Dowden, Hongwu Xu, Yusheng Zhao,* and Quanxi Jia**

Experimental details

Target preparation and film deposition:

To prepare the composite target for pulsed laser deposition of Li_3OCl films, Li_2O (Aldrich, 97% purity) and LiCl powders (Aldrich, 99% purity) with stoichiometric ratios were mixed and ground thoroughly in an Ar-filled glove box. The mixed powders were pressed into a pellet at a pressure of 3000 psi. The pellet was annealed at 350 ^{o}C in a controlled environment (Ar protection) for 4 hours. The XRD pattern in Figure 1a clearly indicates that the annealing process for target preparation does not result in obvious chemical reaction between the raw materials.

The Li_3OCl films were deposited on Li-coated silicon and polished stainless steel substrates by PLD (KrF laser, 248 nm, 20 Hz) under vacuum (1×10^{-5} Torr) using the as-prepared composite target. A protective layer of ZnO was deposited on top of the Li_3OCl films without breaking the vacuum (simply by switching the targets) to prevent Li_3OCl from direct exposure to air during XRD and SEM measurements. The multi-layer films of Au/Li_3OCl/Au and Li/Li_3OCl/Li were deposited without breaking the vacuum to study the ionic conductivity, cyclability, and compatibility with lithium metal of the Li_3OCl films. The detailed PLD parameters for the deposition of different materials are shown in Table S1.

Characterizations and electrochemical measurements:

The structure of the films was studied by X-ray diffraction (XRD, Rigaku Ultima III), and the interfacial characteristic between Li_3OCl and Li metal was investigated by scanning electron microscopy (SEM) instrument equipped with an in-situ Focused Ion Beam (FIB)

system. Electrochemical measurements were carried out using an air-tight cell. AC electrochemical impedance spectroscopy (EIS) measurements were conducted in the frequency range from 100 Hz to 4 MHz with a voltage amplitude of 10 mV by using an electrochemical system (PARSTAT 2273, Princeton Applied Research). The temperature was controlled from room temperature to 140 °C. The ionic conductivities and the activation energy of the Li_3OCl films were derived from the impedance spectra. The symmetric cell of $Li/Li_3OCl/Li$ was cycled by applying a constant current of 1.0 mA with periodically changed polarity using a battery test station (Arbin BT-2000) at room temperature. Full solid-state LIBs with a configuration of graphite/$Li_3OCl/LiCoO_2$ were constructed via a layer-by-layer deposition route, and the cycling tests were carried out between 2.2 and 4.2 V at an applied current of 10 mA g^{-1} at room temperature. The morphologies of graphite anode and $LiCoO_2$ cathode used in the full batteries are shown in Figure S3.

Table S1. PLD parameters used for deposition of different materials. The KrF laser with wavelength of 248 nm was used.

Films	Li_3OCl layer	ZnO layer	Au layer	Li layer	$LiCoO_2$ layer
**Li_3OCl/ZnO**	4.0 J cm^{-2} 30 Hz, 60 min, 175 ^oC, vacuum	4.0 J cm^{-2} 30 Hz, 30 min, 175 ^oC, 50 mTorr Ar	N/A	N/A	N/A
**$Au/Li_3OCl/Au$**	4.0 J cm^{-2} 30 Hz, 60 min, 175 ^oC, vacuum	N/A	6.0 J cm^{-2} 20 Hz, 60 min, 175 ^oC, 50 mTorr Ar	N/A	N/A
**$Li/Li_3OCl/Li$**	4.0 J cm^{-2} 30 Hz, 60 min, 175 ^oC, vacuum	N/A	N/A	2.0 J cm^{-2} 30 Hz, 60 min, 175 ^oC 50 mTorr Ar	N/A
**$C/Li_3OCl/LiCoO_2$**	4.0 J cm^{-2} 30 Hz, 60 min, 175 ^oC, vacuum	N/A	N/A	N/A	5.0 J cm^{-2} 10 Hz, 30 min, 200 ^oC, 75 mTorr O_2
**$Li/Li_3OCl/ZnO$**	4.0 J cm^{-2} 30 Hz, 60 min, 175 ^oC, vacuum	4.0 J cm^{-2} 30 Hz, 30 min, 175 ^oC, 50 mTorr Ar	N/A	2.0 J cm^{-2} 30 Hz, 60 min, 175 ^oC 50 mTorr Ar	N/A

Table S2. Summary of properties and synthetic methods for various solid-state electrolytes.

Electrolytes	σ at RT (S cm^{-1})	E_a (eV)	Working window	Stability with Li	Synthesis methods
Li_3OCl in this work	2×10^{-4}	0.35	> 5 V	Stable	Pulsed laser deposition
Li_3OCl in reference[1]	9×10^{-6}	0.36	> 5 V	Stable	Pulsed laser deposition
LiPON[2-3]	10^{-6}	0.7	1.0–5.0 V	Stable	Sputtering
Single-crystal Li_3N[4]	10^{-3}	0.25	N/A	Stable	Czochralski technique
$Li_{14}Zn(GeO_4)_4$[5]	10^{-6}	0.42	N/A	Unstable	Solid state reaction
$Li_7La_3Zr_2O_{12}$[6]	10^{-4}	0.36	>3V	Stable	Solid state reaction
γ-Li_3PS_4[7]	10^{-7}	0.49	N/A	Unstable	Solid state reaction
β-Li_3PS_4 (nano)[8]	10^{-4}	0.35	> 5 V	Unstable	THF-assisted solution method
Li_4SnS_4[9]	10^{-5}	0.4	> 5 V	Unstable	Solid state reaction

Figures

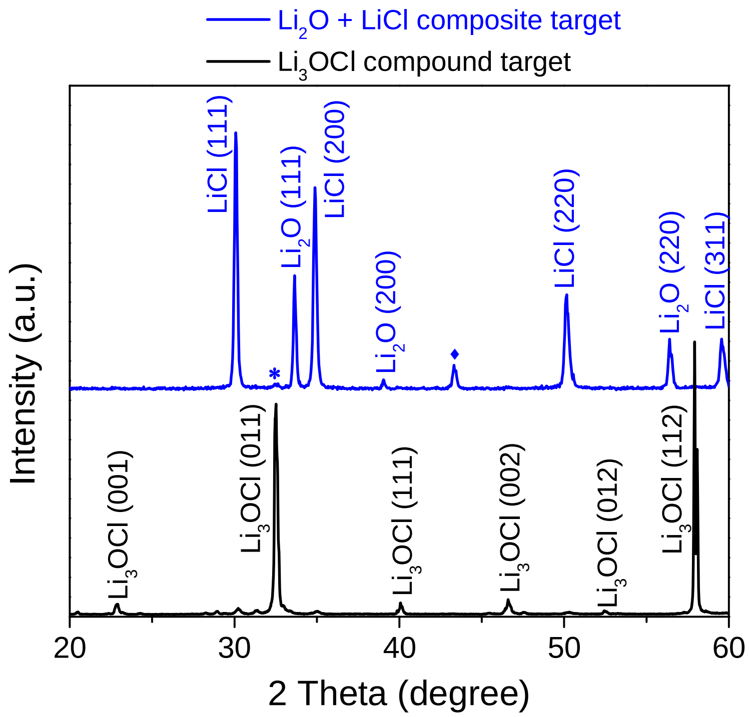

Figure S1. XRD patterns of Li_2O + LiCl composite target and fully reacted Li_3OCl compound target. The symbol of ♦ indicates the peak from Cu holder.

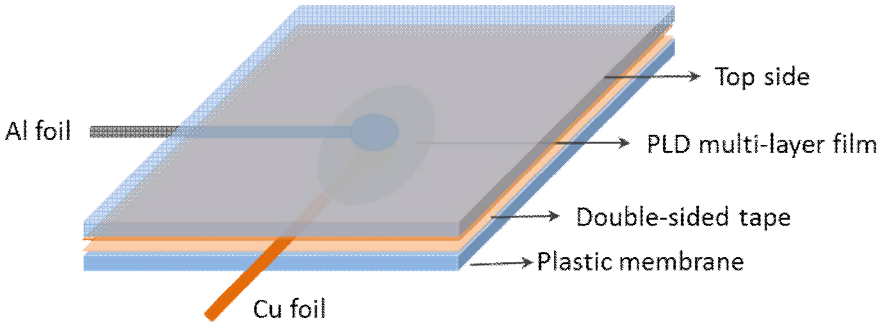

Figure S2. Schematic structure of the membrane-type solid-state Li-ion battery.

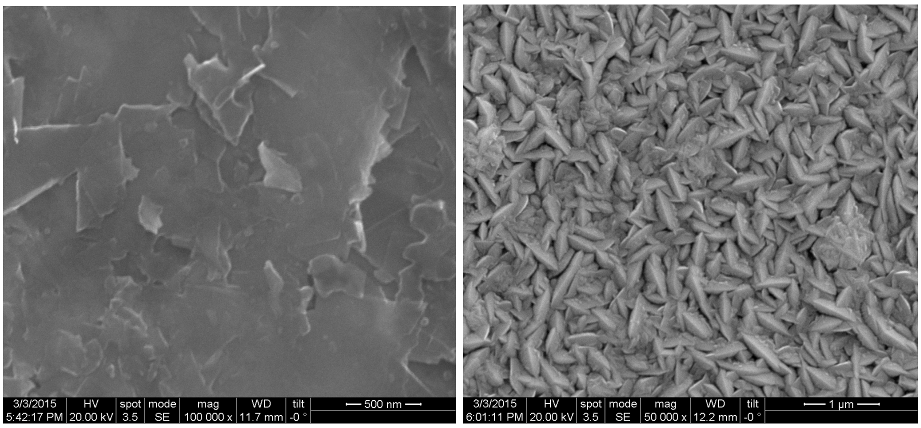

Figure S3. SEM images of graphite anode (left) and $LiCoO_2$ cathode (right) used in the full thin-film LIBs.

References

[1] X. Lü, G. Wu, J. W. Howard, A. Chen, Y. Zhao, L. L. Daemen, Q. Jia, *Chem. Commun.* **2014**, *50*, 11520-11522.

[2] H. Y. Park, S. C. Nam, Y. C. Lim, K. G. Choi, K. C. Lee, G. B. Park, S.-R. Lee, H. P. Kim, S. B. Cho, *J. Electroceram.* **2006**, *17*, 1023-1030.

[3] F. Muñoz, A. Durán, L. Pascual, L. Montagne, B. Revel, A. C. M. Rodrigues, *Solid State Ionics* **2008**, *179*, 574-579.

[4] U. v. Alpen, A. Rabenau, G. H. Talat, *Appl. Phys.Lett.* **1977**, *30*, 621-623.

[5] U. v. Alpen, M. F. Bell, W. Wichelhaus, K. Y. Cheung, G. J. Dudley, *Electrochim. Acta* **1978**, *23*, 1395-1397.

[6] A. Kaeriyama, H. Munakata, K. Kajihara, K. Kanamura, Y. Sato, T. Yoshida, *ECS Transactions* **2009**, *16*, 175-180.

[7] M. Tachez, J.-P. Malugani, R. Mercier, G. Robert, *Solid State Ionics* **1984**, *14*, 181-185.

[8] Z. Liu, W. Fu, E. A. Payzant, X. Yu, Z. Wu, N. J. Dudney, J. Kiggans, K. Hong, A. J. Rondinone, C. Liang, *J. Am. Chem. Soc.* **2013**, *135*, 975-978.

[9] G. Sahu, Z. Lin, J. Li, Z. Liu, N. Dudney, C. Liang, *Energy Environ. Sci.* **2014**, *7*, 1053-1058.
